# Adolescent alcohol, nuts, and fiber: combined effects on benign breast disease risk in young women

**DOI:** 10.1038/s41523-020-00206-4

**Published:** 2020-11-23

**Authors:** Catherine S. Berkey, Rulla M. Tamimi, Walter C. Willett, Bernard Rosner, Martha Hickey, Adetunji T. Toriola, A. Lindsay Frazier, Graham A. Colditz

**Affiliations:** 1 Channing Division of Network Medicine, Department of Medicine, Brigham & Women’s Hospital and Harvard Medical School, Boston, MA USA; 2 Division of Epidemiology, Department of Population Health Sciences, Weill Cornell Medicine, New York, NY USA; 3 Departments of Nutrition and Epidemiology, Harvard T.H. Chan School of Public Health, Boston, MA USA; 4 Department of Obstetrics and Gynaecology, University of Melbourne, Parkville, VIC Australia; 5 Division of Public Health Sciences, Department of Surgery, Washington University School of Medicine and Alvin J Siteman Cancer Center, St. Louis, MO USA; 6 Department of Pediatric Oncology, Dana-Farber Cancer Institute, Boston, MA USA; 7 Alvin J. Siteman Cancer Center, Washington University School of Medicine, St. Louis, MO USA

**Keywords:** Risk factors, Cancer epidemiology

## Abstract

Adolescent drinking is associated with higher risks of proliferative benign breast disease (BBD) and invasive breast cancer (BC). Furthermore, adolescent nut and fiber consumptions are associated with lower risks of benign lesions and premenopausal BC. We hypothesize that diet (nuts, fiber) may mitigate the elevated BBD risk associated with alcohol. A prospective cohort of 9031 females, 9–15 years at baseline, completed questionnaires in 1996–2001, 2003, 2005, 2007, 2010, 2013, and 2014. Participants completed food frequency questionnaires in 1996–2001. In 2005, participants (>=18 years) began reporting biopsy-confirmed BBD (*N* = 173 cases). Multivariable logistic regression estimated associations between BBD and cross-classified intakes (14–17 years) of alcohol and peanut butter/nuts (separately, total dietary fiber). Only 19% of participants drank in high school; drinking was associated with elevated BBD risk (OR = 1.75, 95% CI: 1.20–2.56; *p* = 0.004) compared to nondrinkers. Participants consuming any nuts/butter had lower BBD risk (OR = 0.64, 95% CI: 0.45–0.90; *p* = 0.01) compared to those consuming none. Participants in top 75% fiber intake had lower risk (OR = 0.57, 95% CI: 0.40–0.81; *p* = 0.002) compared to bottom quartile. Testing our hypothesis that consuming nuts/butter mitigates the elevated alcohol risk, analyzing alcohol and nuts combined found that those who consumed both had lower risk (RR = 0.47, 95% CI: 0.24–0.89; *p* = 0.02) compared to drinkers eating no nuts. Our analysis of alcohol and fiber together did not demonstrate risk mitigation by fiber. For high school females who drink, their BBD risk may be attenuated by consuming nuts. Due to modest numbers, future studies need to replicate our findings in adolescent/adult females. However, high school students may be encouraged to eat nuts and fiber, and to avoid alcohol, to reduce risk of BBD and for general health benefits.

## Introduction

During the years prior to a woman’s initial full-term pregnancy, her mammary gland cells are undergoing rapid proliferation making this a critical period for exposures that may increase her lifetime breast cancer risk^1^. Certain exposures from childhood and adolescence confer a higher risk, than those from adulthood, for the development of breast cancer^2–5^, so efforts to prevent disease should begin at early ages^6^. Studies conducted in both animals and humans suggest mechanisms by which in utero childhood and adolescent exposures may impact risks for cancer in women^7–9^.

Benign breast disease (BBD), even without atypia, is a well-established risk factor for breast cancer^10,11^. For this reason, investigating dietary factors in adolescent females and following their subsequent development of BBD may elucidate some origins of breast cancer and may suggest new approaches for its prevention. Among participants in the Nurses’ Health Study II (NHSII), fiber intake during adolescence was inversely associated with risk of proliferative BBD^12,13^, as were nuts^13^. Alcohol intake (ages 18–22 years) was associated with higher risk of proliferative BBD^14^, as was alcohol consumption between menarche and first pregnancy^15^. Analyses of females in the Growing Up Today Study (GUTS) showed lower risk of biopsy-confirmed BBD in young women who had consumed more peanut butter and nuts at age 14 years and also showed a trend (*p* = 0.06) toward higher total dietary fiber consumption (age 14 years) and lower risk of BBD^16^. In this cohort, those (16–23 years) with higher alcoholic beverage consumption had increased risk for BBD^17^.

Taken together, these data suggest that the BBD (and potentially breast cancer) risk associated with adolescent alcohol consumption may be attenuated by increasing adolescent intakes of nuts, peanut butter, or dietary fiber. Longitudinal data from GUTS facilitate the investigation of relationships between adolescent dietary factors and BBD. We consider the combined effect of alcohol and nut/peanut butter consumption (and, separately, total dietary fiber), by girls aged 14–17 years (in high school 1996–2001), on risk of biopsy-confirmed BBD in young women.

## Results

### Participant characteristics

Eighty-two percent of our female participants returned at least one survey (2005–2014) containing questions about BBD. Comparing childhood data of these participants with the non-responding 18%, we found only small differences that are unlikely to create bias in our results. Both groups had similar rates of maternal BBD and maternal breast cancer (both *p* > 0.80). The included females were slightly younger (<5 weeks) at baseline than those not included, but gestational weight gain, age 10 years height, and age 10 years body mass index (BMI) were similar between the two groups. However, those included did have peak height growth velocities that were ¼ inch higher (*p* < 0.05). But more importantly, the two groups had similar age 14-year intakes of alcohol, nuts/peanut butter, and fiber (all *p* > 0.49).

Table 1 presents means (and percentages), within three categories of alcohol intake at ages 14–17 years, of dietary exposure variables and other important characteristics. Girls consuming ≥5 g/day tended to be slightly older at the time of reporting drinking, have slightly lower fiber intakes, had greater maternal gestational weight gain, and were slightly taller at 10 years. They appeared more likely to have mothers with BBD but less likely to have mothers with breast cancer.

**Table 1:** Participant exposures and characteristics within category of alcohol intake from ages 14 through 17 years^a^.

	Alcohol Intake ages 14–17 years		
	None	<5 g/day	≥5 g/day
*N*	5305	966	283
Age (years)	15.20	15.98	16.13
Alcohol ethanol (g/day)	0	1.77	12.02
Nuts/peanut butter (servings/day)	0.18	0.15	0.17
Fiber (g/day, EA)	16.59	16.40	15.25
BMI at 10 years (kg/m^2^)	18.3	18.3	18.3
Height at 10 years (inch)	57.0	57.7	57.9
Gest weight gain (lb)	31.1	31.7	32.6
PHV (inch/year)	3.25	3.19	3.31
Peak age (years)	12.37	12.32	12.3
Age at Menarche (years)	12.89	12.69	12.80
Adult height (inch)	65.4	65.2	65.8
BMI at 18 years (kg/m^2^)	22.5	22.6	22.2
BBD in GUTS (%)	2.21	3.94	2.92
Maternal BBD (%)	25.26	24.12	27.92
Maternal BC (%)	5.47	5.07	2.83

^a^For each girl, her intakes (of alcohol, nuts, and fiber) are all from her year of maximum ethanol consumption reported during that age period.

### Individual exposure models

In Table 2, we present a separate analysis for each of the three adolescent dietary factors to assess the main associations between them and BBD risk. Because Table 1 showed that only 4% of the girls consumed the greatest amounts of alcohol (≥5 g/day, equivalent to approximately 2.5 shots liquor, or 3 servings of wine or beer, per week), our analysis combines intakes >0 into a single group. Girls consuming any alcohol had significantly increased BBD risk (odds ratio (OR) = 1.75, confidence interval (CI): 1.20–2.56; *p* = 0.004) compared to non-drinkers. Girls consuming any nuts/peanut butter were at significantly lower risk (OR = 0.64, *p* = 0.01) compared to those eating neither, and the per serving association was significant (OR = 0.34/daily serving, *p* = 0.016; *P* for trend = 0.01). Girls consuming the least dietary fiber (bottom 25%) appeared to be at significantly greater risk than the 75% consuming larger amounts (OR = 0.57, *p* = 0.002, top 75% compared to bottom 25%). An additional model was estimated that included the three dietary factors (alcohol, nuts, fiber) simultaneously, and there was little moderation of effects compared to Table 2: for alcohol OR = 1.69 (*p* = 0.008), for peanut butter/nuts OR = 0.69 (*p* = 0.035), and for dietary fiber OR = 0.62 (*p* = 0.009). Furthermore, there is no evidence that any one factor would be more clinically relevant (for prevention efforts) than the others.

**Table 2:** Ages 14–17-year intakes of alcohol, peanut butter/nuts, and dietary fiber in separate multivariable^a^ logistic models of risk for biopsy-confirmed BBD (reported at age ≥18 years).

	No. of BBD cases/total *N*	Multivariable OR (95% CI; *p* value)
Alcohol intake (girl’s max from age 14 to 17 years)		
None	101/4565	1.00 (referent)
>0 g/day	39/1051	1.75 (1.20–2.56; 0.004)
Per 10 g/day	1.13 (0.68–1.87; 0.64)	*P*_trend_ = 0.18
Peanut butter and nut intake		
None	55/1678	1.00 (referent)
>0 serving/week	85/3934	0.64 (0.45–0.90; 0.01)
Per serving/day (0–2)	0.34 (0.14–0.82; 0.016)	*P*_trend_ = 0.01
Dietary fiber intake		
Bottom 25%	49/1313	1.00 (referent)
Top 75%	90/4225	0.57 (0.40–0.81; 0.002)
Per 10 g/day	0.68 (0.45–1.03; 0.068)	*P*_trend_ = 0.049

^a^Adjusted for maternal breast cancer, maternal BBD, participant’s age, gestational weight gain, BMI at 10 years, height at 10 years, and adolescent peak height growth velocity.

### Jointly classified exposure models

To test our hypothesis that females who drank alcohol in high school have their risk of BBD mitigated by also consuming nuts and/or peanut butter, we fit a model with alcohol and nut intake jointly classified; the referent group includes females drinking alcohol but consuming no nuts/peanut butter. Girls who ate no nuts but who did drink alcohol had significantly greater BBD risk than all three other groups (*p* = 0.01, *p* = 0.01, and *p* = 0.02; Table 3). Girls who consumed alcohol but who also ate nuts had significantly lower risk (OR = 0.47, CI: 0.24–0.89; *p* = 0.02) than drinkers who never ate nuts (referent), suggesting significant risk mitigation by nut consumption. The *P* for interaction (alcohol group × nut group) was not significant (0.23), so we do not present analyses stratified by nut group. The corresponding analysis of alcohol and dietary fiber (bottom of Table 3) found highest risk among girls drinking alcohol who consumed the least dietary fiber (bottom 25%), though the risk was significantly lower only among those who did not drink and were in top 75% of fiber intake (OR = 0.38; *p* < 0.01). Among drinkers, the BBD risk was suggestively mitigated if they consumed more fiber (top 75%) but the difference was not significant (OR = 0.67, CI: 0.34–1.32; *p* = 0.25, compared to drinkers in bottom quartile fiber). The *P* for interaction (alcohol group × fiber group) was not significant (*p* = 0.63).

**Table 3:** Ages 14–17-year intakes of alcohol (none vs some) jointly classified with same age intakes of nuts/peanut butter (none vs some) in multivariable logistic models of risk for biopsy-confirmed BBD^a^.

Alcohol intake	Nuts/peanut butter	
	None	Some
None	0.44 (0.25–0.79; 0.01)	0.33 (0.19–0.55; <0.01)
	(36/1344)	(65/3217)
Some	1.00 (referent)	0.47 (0.24–0.89; 0.02)
	(19/334)	(20/717)
*P* for interaction = 0.23		

Alcohol intake	Dietary fiber (EA) intake	
	Lowest quartile	Top 3 quartiles
None	0.69 (0.36–1.31; 0.26)	0.38 (0.21–0.69; <0.01)
	(35/1021)	(66/3486)
Some	1.00 (referent)	0.67 (0.34–1.32; 0.25)
	(14/292)	(24/739)
*P* for interaction = 0.63		

^a^Shown within each cell is the odds ratio, 95% CI, *p* value, #BBD cases, and *N*. Because our hypothesis is that, among females consuming alcohol, their risk is lower if they also consume nuts, the reference group is some alcohol and no nuts. The bottom half of this table similarly investigates alcohol combined with same age dietary fiber (lowest quartile vs top 3 quartiles), with reference group any alcohol consumption and least dietary fiber.

## Discussion

A body of previously published evidence indicates that alcohol intake in adolescence increases the risk of BBD and breast cancer, while nuts/peanut butter and dietary fiber reduce those risks. To our knowledge, no other study has investigated whether adolescent consumption of nuts or total dietary fiber may attenuate the increased risk associated with alcohol. Our findings confirmed earlier studies showing that adolescent alcohol consumption was associated with an elevated risk for BBD in young adulthood and that adolescent consumption of nuts and total fiber were separately associated with lower risk. In a model with all three factors together, the associations of each with BBD were nearly as strong as from separate models, and none appeared substantially stronger than the others. Furthermore, we demonstrated that the increased BBD risk associated with alcohol consumption between ages 14 and 17 years was attenuated in females who also consumed any nuts or peanut butter (OR = 0.47, *p* = 0.02). However, for total dietary fiber the estimated risk attenuation (OR = 0.67; *p* = 0.25) was not significant, possibly reflecting difficulties in assessing total dietary fiber from 132 foods on the food frequency questionnaire (FFQ) compared to counting bags of nuts and peanut butter sandwiches.

The age range of intake for our three dietary exposures, 14–17 years, may be a more effective time period for parental (and other adult) influence than older ages, and our findings emphasize the importance of disease prevention messages during high school. Because we found that all three dietary factors were independently strongly associated with BBD risk, and none was substantially stronger than the others, BBD prevention efforts should focus on all three. Given the demonstrated adverse effects of early alcohol consumption, we believe the public health message should emphasize avoiding alcohol in high school, increasing dietary fiber intake, and adding nuts and peanut butter to diet (unless allergies). The message should not be that students may drink if they also consume nuts or peanut butter. Because the lowest BBD risks were among girls who drank no alcohol but consumed nuts or higher levels of fiber, while the highest risks were among girls drinking alcohol but who never ate nuts or consumed the least fiber, avoiding alcohol altogether is still the best strategy for disease prevention.

Significant statistical interaction between the effects of two factors of interest (alcohol and diet) is not required for clinical relevance to BBD prevention. For both our analyses (alcohol and nuts, alcohol and fiber), the main effects were statistically significant, but their interaction was not. If two factors only have additive main effects on BBD risk, it is still relevant that one factor (alcohol) increases risk by a certain amount, while at the same time the other factor (nuts) independently reduces risk by another amount. The effects would off-set each other to some extent, the precise amount dependent upon the magnitudes of the two independent effects, so clinically it would still suggest that increasing consumption of nuts can mitigate the harmful effect of alcohol. In short, the lack of interaction does not rule out their independent main effects, which here are significant and important.

Our findings are consistent with the few published studies on adolescent diet and BBD; the highest quintile of adolescent (high school) fiber intake had 25% lower risk of proliferative BBD than the lowest intake quintile^13^. Adolescent females who consumed ≥2 servings/week of nuts had 36% lower risk of proliferative BBD than those who consumed <1 serving/month^13^. Here our estimated risk of drinking alcohol (OR = 1.13/(10 g/day)) in high school (14–17 years), though not significant, is near the estimated BBD risk of drinking between 18 and 22 years in the NHSII cohort (hazard ratio = 1.15/(10 g/day), CI: 1.03–1.28)^14^. In that cohort, the risk for proliferative BBD from drinking alcohol between menarche and first pregnancy was relative risk = 1.16/(10 g/day) (CI: 1.02–1.32)^15^. Our earlier published work (GUTS cohort) on BBD and drinking at older ages (through 23 years) found significantly increased BBD risk (OR = 1.50 per drink per day; CI: 1.19–1.90)^17^. Furthermore, our findings are consistent with studies of breast cancer, where risks were positively associated with adolescent alcohol^15^ and inversely associated with adolescent consumption of nuts^18^ and fiber^18,19^.

Possible mechanisms, with increased vulnerability between menarche and first pregnancy^20^, include alcohol increasing the levels of circulating estrogen (estradiol and estrone), estrogen receptor (mammary epithelial cells), and carcinogenic ethanol metabolites^21^. Kim et al.^22^ summarize other mechanisms by which alcohol may contribute to increased risk. Regarding possible mechanisms for dietary fiber, as summarized by Farvid et al.^19^, fiber may improve insulin sensitivity, decrease insulin-like growth factors, and decrease plasma levels of estrogen by inhibiting its reabsorption. Unfortunately, our food questionnaire did not ask about different types of nuts, but walnuts contain bioactive molecules affecting mammary epithelial cells and decreasing proliferation^23^. Peanuts and tree nuts provide a broad range of metabolic benefits, attributable to unsaturated fatty acids and other bioactive compounds^24,25^.

Unfortunately, we do not have data on mammographic density, another breast cancer risk factor^10,26^ that may be impacted by diet in high school and represent a different pathway to disease. In NHSII women, adolescent fiber intake was not associated with mammographic density, though there was a marginal (*P* for trend = 0.05) positive association between adolescent nut intake and absolute non-dense breast area; one serving/week of nuts was significantly associated with larger absolute non-dense breast area^27^, which is inversely associated with breast cancer risk. Adolescent alcohol intake was not associated with breast density^28^.

The major strength of our investigation is the longitudinal design, in which alcohol and dietary intakes were reported in real time, years before BBD was reported. Other strengths and weaknesses are similar to those described in previous investigations of this cohort^16^. A limitation is the small number, only 13% of our adolescent females, who consumed both alcohol and nuts, making it difficult to estimate the combined joint effects of specific quantities. A similar small percent consumed both alcohol and fiber above the bottom quartile. Admittedly our cohort is not representative, but this should not hinder the validity and generalizability of our within-cohort comparisons^29^. Because our cohort is 95% white/non-Hispanic, we cannot generalize our results to other races and ethnicities.

In conclusion, we investigated the relationship between biopsy-confirmed BBD in young women and alcohol consumption during adolescence, and the possible attenuation of the alcohol-associated risk by nuts/peanut butter or fiber, during a period critical for the development of breast cancer. Based on girls born in the 1980s and attending high school during 1996–2001, we found evidence that drinking in adolescence (ages 14–17 years) was associated with greater risk of biopsy-confirmed BBD, while same age intakes of nuts/peanut butter and fiber were each associated with lower risk. Among females who drank alcohol in high school, this work found significant risk attenuation by including consumption of nuts and/or peanut butter. For many reasons in addition to reducing risk for BBD, public health interventions should focus on all three factors, reducing alcohol use by adolescents and promoting a healthy diet, which includes consumption of fiber and if no allergies, nuts and peanut butter. Because these findings are based on modest numbers, future studies need to replicate our results in adolescent and/or adult females.

## Methods

### Study population

The GUTS cohort (founding PI, Dr. Colditz) includes 9031 females from 50 states (in the US) who are daughters of participants in the NHSII^30^. This study was approved by the Institutional Review Board at Brigham and Women’s Hospital in Boston. The participants’ mothers provided written informed consent, and their 9–15-year-old daughters assented by completing baseline questionnaires in 1996. The cohort returned questionnaires (by mail on paper or on the Internet) annually beginning in 1996–2001, then in 2003, 2005, 2007, 2010, 2013, and 2014. The response rate for follow-ups (≥1) after baseline is 97%. Most (95%) of our participants are white/non-Hispanic.

### Benign breast disease

Our surveys, beginning in year 2005 and through 2014, inquired “Has a health care provider ever diagnosed you as having benign breast disease?” and whether it had been “Confirmed by breast biopsy”. A total of 7362 participants (when aged 18–32 years) reported that a health care provider had ever (*n* = 385) or never diagnosed them with BBD and whether their diagnosis had been confirmed by breast biopsy (*n* = 173). Six girls, whose mothers reported childhood cancer in them, were excluded from our BBD groups, as were those whose BBD was not confirmed by biopsy. The remaining 6971 females who returned surveys during this period but never reported any BBD diagnosis became our “non-cases” for comparison with those having biopsy-confirmed BBD (*n* = 173).

Most of these cases of biopsy-confirmed BBD were probably diagnosed because participants, or their physicians, found a clinically palpable mass, which was subsequently biopsied. Screening mammography is not offered in this young population. In young women, the most common BBD is fibroadenoma (almost 70% of benign lesions), with the others being mostly cysts and fibrocystic changes^31^. Among women (*N* = 621) in the NHSII, a validation study found 95% accuracy for self-reported biopsy-confirmed BBD^32^.

### Dietary intakes of older children and adolescents

Our research group developed an FFQ, specifically for use on our cohort of older children and adolescents, and showed that it has good validity and reproducibility^33^. The average correlation coefficient for nutrients was 0.54, comparable to that reported in adults^33^. FFQs for adolescents were found, by a meta-analysis, to have good correlations with food records and with 24-h recalls^34^.

Our FFQ inquired about the usual frequency of past year intakes of a wide variety of foods, including 132 questions about foods and beverages commonly consumed by older children in the 1990s. We estimated nut consumption from reports of peanut butter sandwiches and small bags of peanuts or nuts. Our surveys inquired about alcohol consumption during a typical week over the past year of beer, wine, wine coolers, and liquor. We converted servings/day of each to g/day of ethanol using 12.8 g for beer, 11 g for wine, and 14 g for liquor. Alcohol consumption from an FFQ is highly correlated with alcohol consumption from multiple dietary records in women (*r* = 0.90)^35^. The privacy of self-administered questionnaires about risky behaviors in adolescence improves accuracy for reported alcohol consumption in adolescents^36^. Test–retest reliability levels were generally high for alcohol, but biochemical validation of alcohol consumption was not available^36^. Total fiber and total energy were calculated based on all food and beverage intakes. Our analyses use energy-adjusted (residual-method) fiber.

Here we use FFQs from the 1996, 1997, 1998, and 2001 surveys to obtain dietary data at ages 14–17 years. A total of *N* = 6554 girls provided alcohol intakes between the ages of 14 and 17 years. For our analyses, we determined the age (14, 15, 16, or 17 years) when each girl reported drinking the most alcohol (in g/day), and we used her nut and fiber intakes from the same age.

### Other variables

Ages were computed using birth dates and dates when questionnaires were returned. We previously described how we derived those adjustment factors related to child growth that were associated with risk for BBD: maternal weight gain during pregnancy with this child, the child’s height and BMI at age 10 years, and her adolescent peak height growth velocity (PHV)^37^. Weight (and BMI) at age 18 years did not use reported weights of females who were pregnant. Their mothers reported, through year 2013, their own diagnoses of breast cancer and BBD (including biopsy confirmed).

### Statistical analysis

We used logistic regression models, estimated by SAS^38^, to obtain associations between high school dietary factors and biopsy-confirmed BBD in young adulthood. We adjusted all multivariable models for participant’s age at cohort initiation (in 1996), maternal breast cancer and maternal BBD, and four body size factors from childhood that we previously found were important BBD risk factors (maternal weight gain during pregnancy with this daughter, her height and BMI at age 10 years, and her PHV)^37^. We initially obtained three separate models of risk for BBD, each using only one of the adolescent exposures: intakes of alcohol, peanut butter/nuts, or dietary fiber. Each dietary exposure factor was modeled as a categorical variable and separately as a continuous variable (servings/day or g/day). *P* for trend was derived using the median value within each quintile. A subsequent model included alcohol together with nuts/peanut butter; combined effects were estimated using jointly classified categories. The significance of any interaction between nuts and alcohol was assessed using a cross-product term (alcohol × nuts, both categorical, in a model including main effects for alcohol and nuts). For estimating the combined effects of alcohol and dietary fiber, the same approach (outlined above) was used. All statistical tests are two sided.

### Reporting summary

Further information on research design is available in the Nature Research Reporting Summary linked to this article.

## Supplementary information

Reporting Summary Checklist

## Data Availability

The data that support the findings of this study are not publicly available but can be made available to researchers on reasonable request. External investigators, who are interested in collaborating and using the cohort data, are invited to fill out a form that asks about the details of the collaboration. Please visit http://nhs2survey.org/gutswordpress/index.php/researchers/information-for-researchers/ for more information on data access. External collaborators (after being granted permission) will be provided a login and password to get access to the data; the Channing computing system; and detailed documentation and training materials for where to find data, what analytic tools are available, and examples of analyses. The data generated and analyzed during this study are described in the following metadata record^39^: 10.6084/m9.figshare.12957515.
