# Supplementary material for: Adolescent alcohol, nuts, and fiber: combined effects on benign breast disease risk in young women
Source: NPJ Breast Cancer. 2020 Nov 23;6:61. doi: 10.1038/s41523-020-00206-4 (PMC7683739; doi:10.1038/s41523-020-00206-4)
Supplement: Supplementary file 1 — Reporting Summary Checklist [file 41523_2020_206_MOESM1_ESM.pdf]

nature research

Corresponding author(s): Dr. Catherine S. Berkey

Reporting Summary

Nature Research wishes to improve the reproducibility of the work that we publish. This form provides structure for consistency and transparency in reporting. For further information on Nature Research policies, see Authors & Referees and the Editorial Policy Checklist.

Statistical parameters

When statistical analyses are reported, confirm that the following items are present in the relevant location (e.g. figure legend, table legend, main text, or Methods section).

n/a	Confirmed	
☐	☒	The exact sample size (*n*) for each experimental group/condition, given as a discrete number and unit of measurement
☐	☒	An indication of whether measurements were taken from distinct samples or whether the same sample was measured repeatedly
☐	☒	The statistical test(s) used AND whether they are one- or two-sided<br>*Only common tests should be described solely by name; describe more complex techniques in the Methods section.*
☐	☒	A description of all covariates tested
☒	☐	A description of any assumptions or corrections, such as tests of normality and adjustment for multiple comparisons
☐	☒	A full description of the statistics including central tendency (e.g. means) or other basic estimates (e.g. regression coefficient) AND variation (e.g. standard deviation) or associated estimates of uncertainty (e.g. confidence intervals)
☐	☒	For null hypothesis testing, the test statistic (e.g. *F*, *t*, *r*) with confidence intervals, effect sizes, degrees of freedom and *P* value noted<br>*Give P values as exact values whenever suitable.*
☒	☐	For Bayesian analysis, information on the choice of priors and Markov chain Monte Carlo settings
☒	☐	For hierarchical and complex designs, identification of the appropriate level for tests and full reporting of outcomes
☐	☒	Estimates of effect sizes (e.g. Cohen's *d*, Pearson's *r*), indicating how they were calculated
☒	☐	Clearly defined error bars<br>*State explicitly what error bars represent (e.g. SD, SE, CI)*

Our web collection on statistics for biologists may be useful.

Software and code

Policy information about availability of computer code

Data collection	Paper surveys mailed to participants beginning in 1996, and in later years participants could either return the paper survey by mail or complete their survey online.
Data analysis	SAS proc means, proc freq and proc logistic

For manuscripts utilizing custom algorithms or software that are central to the research but not yet described in published literature, software must be made available to editors/reviewers upon request. We strongly encourage code deposition in a community repository (e.g. GitHub). See the Nature Research guidelines for submitting code & software for further information.

Data

Policy information about availability of data

All manuscripts must include a data availability statement. This statement should provide the following information, where applicable:
- Accession codes, unique identifiers, or web links for publicly available datasets
- A list of figures that have associated raw data
- A description of any restrictions on data availability

Researchers interested in access to data from this cohort are referred to

http://nhs2survey.org/gutswordpress/index.php/researchers/information-for-researchers/

Field-specific reporting

Please select the best fit for your research. If you are not sure, read the appropriate sections before making your selection.

☐ Life sciences ☒ Behavioural & social sciences ☐ Ecological, evolutionary & environmental sciences

For a reference copy of the document with all sections, see nature.com/authors/policies/ReportingSummary-flat.pdf

Behavioural & social sciences study design

All studies must disclose on these points even when the disclosure is negative.

Study description	Longitudinal (1996-2014) observational study of quantitative data.
Research sample	Cohort of daughters of Nurses Health Study II participants, age 9-15yr at baseline in 1996, from all over the US. A convenience sample so not representative. We believe this is the only cohort providing the data needed to test our hypothesis.
Sampling strategy	This is a convenience sample, with all the mothers in NHSII asked (in 1995) if they had children in our desired age range. We enrolled every child, in the correct age range, whose mothers provided informed consent, and the child assented by completing and returning the surveys. The cohort currently includes over 9,000 females, who have provided data for manuscripts covering a wide range of topics.
Data collection	Data collection began in 1996 using paper surveys mailed to all participants. In later years they could also complete them online, or still on paper. All the data were self-reported (probably in their homes), and no researchers were present, though their mothers may have helped them, especially with self-reporting heights. Researchers were blind (to disease outcome and to our hypothesis) throughout data collection that occurred many years before we became interested in this research topic.
Timing	First survey in 1996, then 1997, 1998, 1999, 2000, 2001, 2003, 2005, 2007, 2010, 2013 and 2014. Participants in high school 1996-2001.
Data exclusions	We excluded females who had childhood cancer (reported by their mothers) and females who reported they had the disease BBD but it was not confirmed by breast biopsy.
Non-participation	Only 18% of the cohort was lost to follow-up before year 2005 when our surveys began asking about diagnosis of the disease BBD.
Randomization	There was no allocation to experimental groups.

Reporting for specific materials, systems and methods

Materials & experimental systems

n/a	Involved in the study	
☒	☐	Unique biological materials
☒	☐	Antibodies
☒	☐	Eukaryotic cell lines
☒	☐	Palaeontology
☒	☐	Animals and other organisms
☐	☒	Human research participants

Methods

n/a	Involved in the study	
☒	☐	ChIP-seq
☒	☐	Flow cytometry
☒	☐	MRI-based neuroimaging

Human research participants

Policy information about studies involving human research participants

Population characteristics	Females, aged 9-15 at study initial in 1996, residing all over the US. Those who were cancer-free as children were followed to see who developed benign breast disease as young women. Most (95%) were white/NH.
Recruitment	All participants were daughters of women in the Nurses Health Study 2, who confirmed that they had children in our age range of interest, and they provided to us the names of their children and gave us permission to contact them and to enroll them in our study. (Our primary interest back in 1996 was in studying the development of obesity during adolescence.) Though this is not a representative sample of children from across the US, the comparison of risks within our cohort should still be valid.
